# Prenatal Diagnosis of an Intrathoracic Left Kidney Associated with Congenital Diaphragmatic Hernia: Case Report and Systematic Review

**DOI:** 10.3390/jcm12113608

**Published:** 2023-05-23

**Authors:** Giuliana Orlandi, Paolo Toscano, Olimpia Gabrielli, Enrica Di Lella, Antonia Lettieri, Luigi Manzo, Laura Letizia Mazzarelli, Carmine Sica, Letizia Di Meglio, Lavinia Di Meglio, Ferdinando Antonio Gulino, Giosuè Giordano Incognito, Attilio Tuscano, Stefano Cianci, Aniello Di Meglio

**Affiliations:** 1 Department of Neuroscience, Reproductive Sciences and Dentistry, School of Medicine, University of Naples Federico II, 80131 Naples, Italy; giulianaorlandi@msn.com (G.O.); paol.toscano@gmail.com (P.T.); enrica_dilella@hotmail.it (E.D.L.); luigimanzo93@libero.it (L.M.); lauramazzarelli@gmail.com (L.L.M.); 2 Diagnostica Ecografica e Prenatale di A. Di Meglio, 80133 Naples, Italy; antonia_lettieri@libero.it (A.L.); sicacarmine111@gmail.com (C.S.); aniellodimeglio@gmail.com (A.D.M.); 3 Radiology Department, School of Medicine, University of Milan, 20133 Milan, Italy; letiziadimeglio@gmail.com; 4 Pediatric Department, Bambino Gesù Children’s Research Hospital IRCCS, 00165 Rome, Italy; laviniadimeglio@gmail.com; 5 Department of Obstetrics and Gynaecology, Azienda di Rilievo Nazionale e di Alta Specializzazione (ARNAS) Garibaldi Nesima, 95124 Catania, Italy; 6 Department of General Surgery and Medical Surgical Specialties, University of Catania, 95123 Catania, Italyattiliotuscano@gmail.com (A.T.); 7 Department of Human Pathology of Adult and Childhood “G. Barresi”, University of Messina, 98121 Messina, Italy; stefanoc85@hotmail.it

**Keywords:** ectopic kidney, congenital diaphragmatic hernia, prenatal diagnosis, congenital malformation, case report

## Abstract

Introduction: A congenital intrathoracic kidney (ITK) is a rare anomaly that is recognized to have four causes: renal ectopia with an intact diaphragm, diaphragmatic eventration, diaphragmatic hernia, and traumatic diaphragmatic rupture. We report a case of a prenatal-diagnosed ITK related to a congenital diaphragmatic hernia (CDH) and conducted a systematic review of all cases of the prenatal diagnosis of this association. Case presentation: A fetal ultrasound scan at 22 gestational weeks showed left CDH and ITK, hyperechoic left lung parenchyma, and mediastinal shift. The fetal echocardiography and karyotype were normal. Magnetic resonance imaging at 30 gestational weeks confirmed the ultrasound suspicion of left CDH in association with bowel and left kidney herniation. The fetal growth, amniotic fluid, and Doppler indices remained within the normal range over time. The woman delivered the newborn via an at-term spontaneous vaginal delivery. The newborn was stabilized and underwent non-urgent surgical correction; the postoperative course was uneventful. Conclusions: CDH is the rarest cause of ITK; we found only eleven cases describing this association. The mean gestational age at diagnosis was 29 ± 4 weeks and 4 days. There were seven cases of right and four cases of left CDH. There were associated anomalies in only three fetuses. All women delivered live babies, the herniated kidneys showed no functional damage after their surgical correction, and the prognosis was favorable after surgical repair. The prenatal diagnosis and counseling of this condition are important in planning adequate prenatal and postnatal management in order to improve neonatal outcomes.

## 1. Introduction

Congenital intrathoracic kidney (ITK) is a rare malformation representing partial or complete protrusion of the kidney above the level of the diaphragm into the mediastinum. This pathological abnormality represents <5% of all renal ectopias. It is more common in the male sex than in the female sex (2:1), and it has a slight left-side predominance.

In most cases, a congenital intrathoracic kidney is asymptomatic and diagnosed incidentally, often after birth; however, it can be misdiagnosed as pneumonia because of its presentation on a chest X-ray as an opacity or lobar consolidation [1,2].

An ITK is recognized as having four main causes: “real” renal ectopia with an intact diaphragm, diaphragmatic eventration, congenital or acquired diaphragmatic hernia, and traumatic diaphragmatic rupture [3].

Congenital diaphragmatic hernia (CDH) is frequently associated with gastric, bowel, or hepatic herniation, whereas renal protrusion is extremely rare.

We report a case of a prenatal diagnosed ITK associated with CDH.

**Figure 1:**
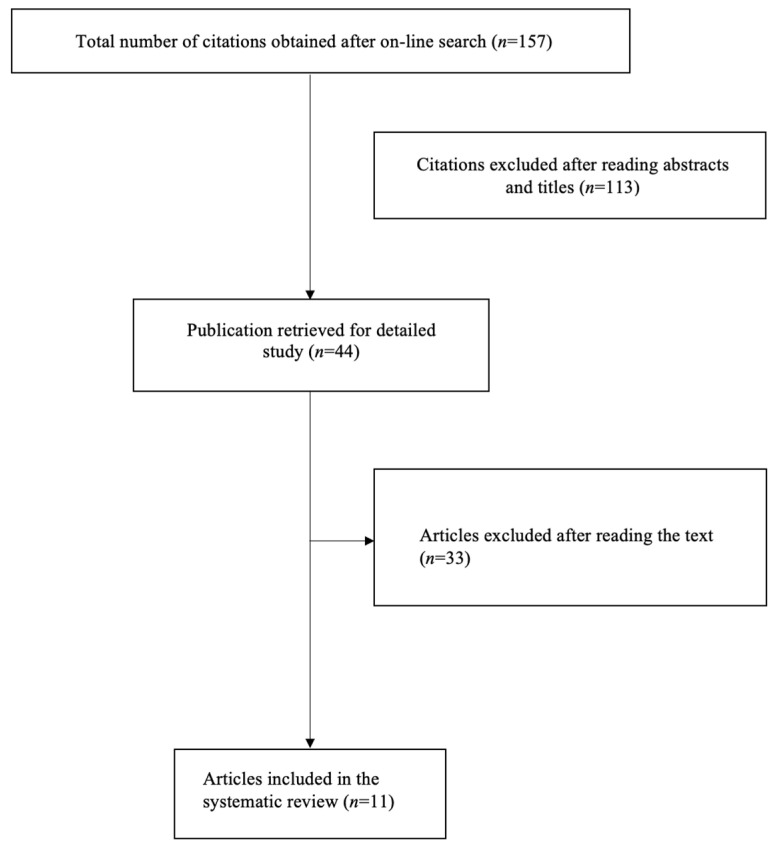
Selection process for the inclusion of suitable studies for the systematic review.

We also conducted a systematic review of all cases of this association diagnosed antenatally using MEDLINE, EMBASE, Scopus, ClinicalTrials.gov, OVID, and the Cochrane Library as electronic databases from January 1970 to December 2022. We used the medical subject heading (MeSH) term Kidney (MeSH Unique ID: D007668) in combination with Hernias, Diaphragmatic, and Congenital (MeSH Unique ID: D065630). No restrictions concerning language or geographic location were applied. The systematic review was performed in accordance with the Preferred Reporting Items for Systematic Reviews and Meta-Analyses (PRISMA) guidelines [4] (Figure 1). One author (F.A.G.) independently screened the titles and abstracts of each citation, after which they selected relevant ones for a full-text review. Each retrieved full-text article was independently evaluated for inclusion by another author (G.G.I.). Any potential disagreement was solved via a discussion with a third author (A.D.M.). After reading the abstracts and titles, 113 articles were excluded because they were not pertinent to the field; 33 articles were excluded after reading the text because the diagnosis of an intrathoracic left kidney was performed in the postnatal period.

## 2. Case Presentation

**Figure 2:**
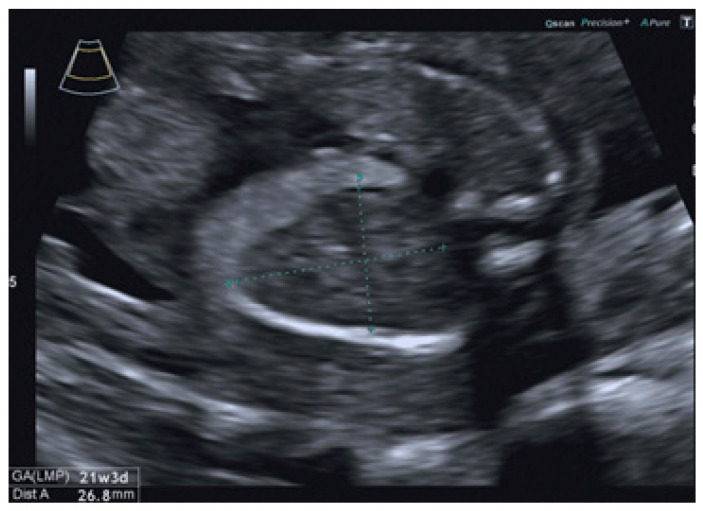
Ultrasound scan at 22 gestational weeks showing an intrathoracic left mass and hyperechoic left lung parenchyma.

A 37-year-old Caucasian woman, gravida 2 para 0, with a history of a previous miscarriage due to an unknown cause, was referred to our second-level center at 22 gestational weeks for a suspicious fetal intrathoracic mass and left CDH at a second-trimester ultrasound (US) screening. Her previous medical and family histories were unremarkable; she was a nonsmoker, had not consumed any alcohol during the pregnancy, and had never been exposed to drugs or toxins. The measurements of the dating examination in the first trimester were consistent with the dates of the last menstrual period, the nuchal translucency was normal, and toxoplasmosis, other agents, rubella, cytomegalovirus, and herpes simplex (TORCH) screening was negative. Our US examination was performed via the use of a Voluson E10 scanner (GE Healthcare Ultrasound, Milwaukee, WI, USA) equipped with a curved linear array transabdominal transducer (2–5 MHz). The fetal heart rate was within the normal range, fetal movements were visualized, the placenta was identified on the anterior portion of the uterus and showed a normal insertion, and the amniotic fluid, as well as the Doppler indices, were within normal limits. The fetal biometry was consistent with the gestational age (GA). An intrathoracic left hypoechoic mass with a maximum diameter of 26.8 mm was seen (Figure 2).

**Figure 3:**
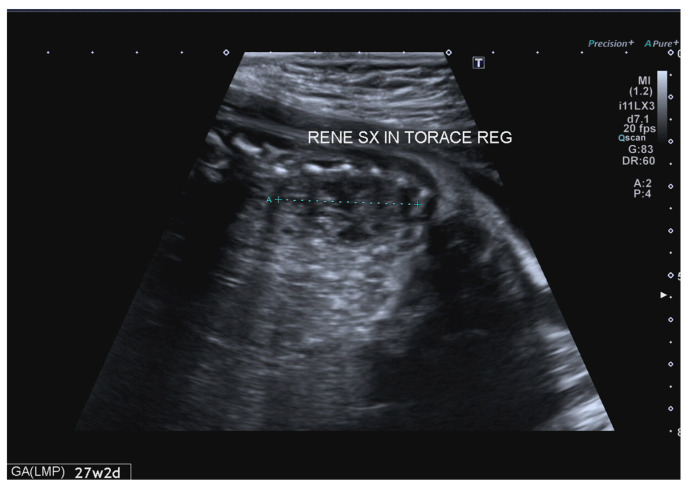
Sagittal view showed a left kidney in the thorax.

The heart was displaced to the right (mediastinal shift), in normal levocardia. Furthermore, the left renal fossa was empty. In the left parasagittal view, the left kidney appeared lifted up toward the thorax (Figure 3).

**Figure 4:**
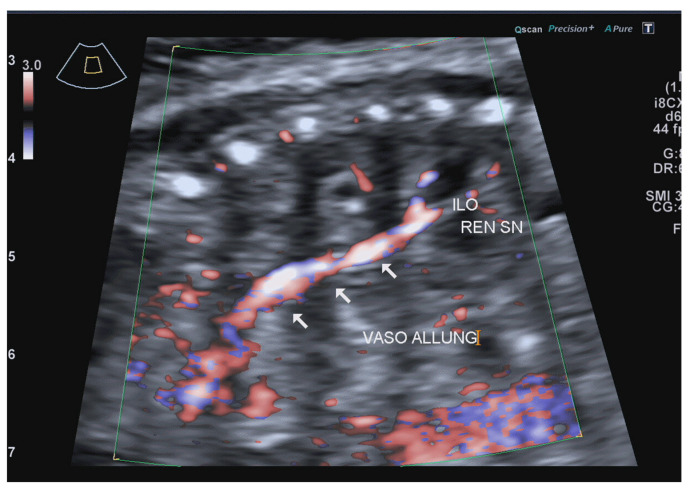
Power Doppler showed the course of the left renal artery, from the abdominal aorta toward the thorax and the renal hilum.

Power Doppler showed the abnormal course of the left renal artery, which started from the abdominal aorta and ended in the thoracic kidney (Figure 4).

The scan did not show a clear herniation of the stomach, bowel, or liver. The lung-to-head circumference ratio (LHR) was greater than 1.4, and the observed-to-expected (o/e) LHR was 52.1%. The left lung parenchyma beside the mass was hyperechoic (Figure 2). The left renal biometry and echogenicity were normal, and no pyelectasia was found (Figure 3). The right kidney was normal, and no other anomalies were identified. Fetal echocardiography verified normal conotruncal anatomy, normal left and right ventricular cavity size as well as systolic function, and sinus rhythm with 1:1 atrioventricular conduction. Our clinical suspicion was of a left CDH and ITK. Furthermore, we suspected an association with a congenital cystic adenomatoid malformation. The patient underwent amniocentesis, and the traditional karyotype as well as comparative genomic hybridization (CGH) array showed a normal female karyotype (46, XX). Subsequently, prenatal management included serial US examinations, including biophysical profile scoring, to monitor fetal growth and assess the fetal heart. On subsequent scans, no changes in the US characteristics of the ectopic kidney were found, and the fetal growth, amniotic fluid, and Doppler indices remained within the normal range for GA. The fetus did not develop hydrothorax. The pregnant woman was referred for fetal magnetic resonance imaging (MRI) at 30 gestational weeks, which confirmed the US suspicion of left CDH in association with bowel and left kidney herniation. After 34 weeks of gestation, cardiotocography was performed weekly to monitor fetal wellbeing, which remained good. The patient was referred to a third-level center for delivery. A female infant weighing 3100 g was delivered via a spontaneous vaginal delivery at 37 weeks of gestation; her Apgar scores at 1 and 5 min were 8 and 9, respectively. The neonate cried immediately after birth, and she was pink and well-perfused. Nevertheless, she developed respiratory distress in the delivery room and required intubation. Chest radiography confirmed the prenatal suspicion of CDH, including bowel loops and the left kidney, with a mediastinal shift of the heart. Echocardiography showed normal heart function and morphology, with no pulmonary hypertension. Due to the hemodynamic stability, the newborn underwent non-urgent surgery on her second day of life through a transverse laparotomy. Surgical exploration confirmed the left kidney with the hernia sac. The organs herniated in the thoracic cavity included small bowel loops, the colon, and the left kidney. The intrathoracic ectopic right kidney was covered with retroperitoneum. After the hernia sac was excised, the small intestine, colon, and ITK were reduced with no difficulties into the abdominal cavity, in a near-to-normal site, without complication or hemodynamic changes. The defect of the diaphragm was primarily repaired using interrupted non-absorbable sutures. A chest tube was placed in the left hemithorax and removed on day four. The corrected kidney showed regular function, with a slight biometric reduction. The postoperative course was uneventful; the baby was extubated on day two and discharged from the hospital after about two months in good condition. She has remained asymptomatic at subsequent follow-ups 1 year after discharge, with normal physical as well as mental development and without any long-term complications, not requiring further monitoring.

## 3. Discussion

A congenital ectopic kidney is a rare malformation, caused by the malposition of the kidney during embryogenesis. Most ectopic kidneys are found in the pelvic and lumbar regions secondary to failure to ascend during fetal life. ITK is extremely rare, with a prevalence ranging from 0.5 to 5% and an incidence of 1 in every 10.000 cases of an ectopic kidney [1,2]. Males (63%) are affected more frequently than females (37%) [5]. It is found more frequently on the left than on the right side, and it is rarely bilateral [5]. It is considered a sporadic malformation and is not associated with an increased risk of recurrence. ITK may be differentiated from other intrathoracic and mediastinal masses using various diagnostic methods, including contrast-enhanced computed tomography scans and MRI. Differential diagnoses should include esophageal duplication cysts, bronchogenic cysts, microcystic adenomatoid malformation, bronchopulmonary sequestration, mediastinal teratoma, and aneurysm of the descending aorta [6]. ITK is recognized as having four main causes: “real” renal ectopia with an intact diaphragm, diaphragmatic eventration, congenital or acquired diaphragmatic hernia, and traumatic diaphragmatic rupture [3]. The “real” variant represents less than 5% of ectopic kidneys [7]. It possesses the following characteristics: rotation anomaly, elongated ureter, and vascularization from the thoracic aorta. Generally, it is asymptomatic, and, if not detected during the antenatal period, may remain silent for many years [8] and require no treatment [9]. Diaphragmatic eventration is a congenital anomaly related to a phrenic nerve injury that causes half or total diaphragm elevation, with an apparent ascent of abdominal viscera into the thorax and no diaphragm defect [10]. The incidence of diaphragmatic eventration is less than 0.05% [11].

CDH is a rare anomaly that affects 2.3–2.8 per 10,000 live births, with a male predominance [12]. The development of the musculotendinous diaphragm, which occurs from the 4th to 12th week of development, involves four embryologic structures: the septum trasversum, the pleuroperitoneal membranes, the mediastinum (dorsal mesentery of the esophagus), and body wall muscles. The gradual fusion of the pleuroperitoneal membranes and septum transversum starts during the fourth week of development [13]. At approximately the eighth week, the closure of the pleuroperitoneal canals occurs when the septum transversum fuses with the structures surrounding the esophagus, the esophageal mesentery, and connects to the pleuroperitoneal membranes [14]. The right and left pleuroperitoneal membranes close the communication between the pleural and peritoneal cavities. A delayed closure or the maldevelopment of the septum transversum and the two pleuroperitoneal folds leads to a diaphragmatic defect, with herniation of abdominal viscera into the thorax [9]. An alternative hypothesis is that lung hypoplasia may be the primary causal factor in the development of CDH [15,16]; it has also been reported in association with the maternal administration of medications such as thalidomide or antiepileptics [17]. The most frequent is a Bochdalek hernia, with a postero-lateral defect, described in 70–95% of CDH cases [10]. Right-sided CDHs are less common [18], due to either the presence of the liver on the right as a physical thoraco-abdominal barrier or the early fusion of the pleura-peritoneal channel on the right side [19]. CDH is usually diagnosed antenatally, the overall prenatal detection rate is 46–52% [20], and the average GA at diagnosis ranges from 24 to 25 weeks [21]. The prenatal diagnosis of CDH is quite straightforward, with echogenic contents seen in the thorax and a mediastinal shift; however, when the kidney is the only herniating organ it can be misdiagnosed as renal agenesis [14]. These signs are also typical of pulmonary sequestration [22]. Using the color Doppler to trace the aberrant renal artery arising from the aorta and coursing upward can help reach the correct diagnosis. In 10–30% of cases, there are associated chromosomal anomalies. The most common include trisomy 12, 18, and 21, Turner syndrome, partial trisomy 5, partial trisomy 20, tetraploidy 21, and tetrasomy 12p [23]. Fetal syndromes, such as Apert, Beckwith–Wiedemann, Coffin-Siris, and Pierre Robin, as well as many others, can be associated with CDH [24,25]. Structural defects are found in 25–57% of all cases [23], and cardiac defects worsen the prognosis most frequently among them (11–15%) [24]. Proof of this degraded prognosis was confirmed through research conducted by Graziano et al. [25], in which 2636 pediatric CDH patients from 82 centers were followed. The survival rate among CDH patients without any heart defect was 70%, whereas those patients with diagnosed heart defects had a significantly lower survival rate of 41.1%. These data were validated by Menon et al. [26], who found the survival rate of infants born with CDH and no heart defects to be 69%; for those with a heart defect, the survival rate was 36%.

Renal herniation related to CDH is rare, with eleven cases [27,28,29,30,31,32,33,34,35,36,37] described in the literature (Table 1).

The mean maternal age was 30 ± 4 years. The mean GA at diagnosis was 29 ± 4 weeks and 4 days. The incidence of renal herniation in CDH was higher on the right side, with seven cases of right [28,29,30,31,35,36,37] and four cases of left CDH [27,32,33,34] reported. A karyotype with CGH array should be offered because of the association with chromosomal and genetic anomalies. None of the cases previously reported and the present one had any chromosomal abnormalities or associated genetic syndromes, although the fetal karyotype has only been determined in three previous cases [28,32,33]. Ultrasound is a fundamental diagnostic tool for the diagnosis of numerous obstetric and gynecological conditions [38,39,40,41,42], and in this case as well, an accurate fetal anatomy study must be offered to exclude associated anomalies. Fetal echocardiography is essential in assessing fetal heart function and structure, as well as in aiding in the detection of pulmonary hypertension and/or pulmonary hypoplasia, and it plays a vital role in prognosis prediction [18]. Associated anomalies have previously been described in three fetuses: one bronchopulmonary sequestration [30], one bicuspid aortic valve [34], and one hepatic pulmonary fusion [35]. Another feature of the present case is the presence of hyperechoic pulmonary parenchyma beside the herniated mass. These findings cannot exclude a concurrent congenital lung cystic adenomatoid malformation. The postnatal surgical evaluation confirmed a compressive effect. This suggests that, in the case of a huge CDH, pulmonary hyperechogenicity is more likely related to a compressive effect, rather than a congenital lung cystic adenomatoid malformation. A fetal MRI was never performed in any of the previous cases, despite its ability to provide further insights into numerous obstetric conditions [43]; this fact aside, it can be useful for the quantitative evaluation of fetal lungs and, in association with the US, can help to predict the neonatal outcomes of prenatally diagnosed CDH, such as total fetal lung volume (TFLV) estimation [44]. As with any case of antenatally diagnosed CDH, the o/e LHR offers the best positive predictive values for postnatal survival [45]. All women included in the systematic review delivered a live baby. The mean GA at delivery was 38 ± 2 weeks. Most cases of ITK reported in the literature have been incidental diagnoses in asymptomatic individuals who may not require any treatment [14]. When isolated, it is an innocuous condition that does not need further extensive investigation [46]; however, the presence of CDH and the associated herniation of abdominal viscera can cause respiratory distress, necessitating surgical repair [14]. According to the previous literature, the present case shows that the herniation of the kidney does not represent the worst prognosis factor for CDH. Indeed, the kidney is not usually damaged from the herniation, preserving its physiological functions, and the prognosis is favorable after surgical repair, as seen in our case.

Improvements in surgical techniques and perioperative care have led to better outcomes for patients with these conditions. For example, minimally invasive surgical approaches, such as laparoscopy and thoracoscopy, have reduced the morbidity associated with open surgery and improved patient recovery times. Similarly, advances in neonatal respiratory support, including extracorporeal membrane oxygenation (ECMO), have increased survival rates for infants with severe CDH. Overall, the implications of these advancements for clinical practice and patient outcomes are significant. Early and accurate diagnosis, as well as effective surgical and perioperative care, can greatly improve the prognosis for patients with an intrathoracic kidney and CDH. As such, continued research and innovation in this field are essential for further improving the outcomes and quality of life of affected individuals.

## 4. Conclusions

ITK associated with CDH is a rare malformation. An accurate study of fetal anatomy is necessary to exclude associated anomalies, and a fetal invasive karyotype must be offered. Fetal MRI can be complementary to the US for prognostic evaluation and differential diagnosis. Renal function is usually preserved, and the prognosis is favorable after surgical repair. In line with prior research, this case report underscores the favorable prognosis for fetuses with kidney herniation, offering guidance to obstetricians for the counseling of this uncommon anomaly. The differential diagnosis of the reported case is a highly involved undertaking that requires a broad spectrum of specialized healthcare professionals. Prenatal counseling should involve a multidisciplinary team involving maternal-fetal medicine, pediatric surgery, genetics, and neonatology. The long-term outlook varies considerably and is based on associated anomalies, preterm delivery, hernia position, and fetal lung volume.

## Figures and Tables

**Table 1:** Studies included in the systematic review.

Study, Year	Country	Maternal Age (Years)	GA at Diagnosis (Weeks)	Side of CDH; Herniated Organs	Associated Anomalies	LHR o/e LHR	Karyotype	GA at Delivery (Weeks) and Mode of Delivery	Newborn Gender	Kidney Damage	Outcome
Singh et al., 2020 [27]	India	30	31	Left; small bowel, transverse colon, and left kidney	None	LHR 3.0; o/e 100%	NA	37 CS	F	None	Favorable after surgical repair
Masturzo et al., 2001 [28]	UK	30	33	Right; bowel, right lobe of the liver, and right kidney	None	NA	Normal	35 VD	M	None	Favorable after surgical repair
Juricic et al., 2015 [29]	France	35	33	Right; small bowel, transverse colon, and right kidney	None	NA	NA	39 VD	F	None	Favorable after surgical repair
Park, 2020 [30]	South Korea	31	28	Right; small intestine, colon, right lobe of the liver, and right kidney	Bronco- pulmonary sequestration	NA	NA	40 VD	F	None	Favorable after surgical repair
Thompson et al., 2019 [31]	USA	33	35	Right; small bowel, liver, and right kidney	None	NA	NA	39 CS	F	None	Favorable after surgical repair
Athanasiadis et al., 2011 [32]	Greece	25	22	Left; spleen, small intestine, and left kidney	None	NA	Normal	34 CS	M	None	Favorable after surgical repair
Hidaka et al., 2012 [33]	Japan	26	28	Left; stomach, small intestine, spleen, and left kidney	None	LHR 1.6	Normal	38 CS	M	None	Favorable after surgical repair
Panda et al., 2009 [34]	USA	23	28	Left; small intestine, spleen, and left kidney	Bicuspid aortic valve (postnatal)	LHR 1.6	NA	40 VD	M	None	Favorable after surgical repair
Takezoe et al., 2017 [35]	Japan	NA	33	Right; right lobe of the liver, right kidney	Hepatic- pulmonary fusion	NA	NA	39 NA	F	None	Favorable after surgical repair
Jeong et al., 2016 [36]	Korea	29	32	Right; right kidney	None	LHR 2.54	Normal	39 VD	F	None	Favorable after surgical repair
Cessans et al., 2015 [37]	France	35	22	Right; small bowel, colon, and right kidney	None	NA	NA	39 VD	F	None	Favorable after surgical repair

Abbreviations: CDH, congenital diaphragmatic hernia; CS, cesarean section; GA, gestational age; LHR, lung-to-head circumference ratio; o/e, observed to expected; NA, not applicable; and VD, vaginal delivery.

## Data Availability

Not applicable.
